# Calorie Restriction Attenuates Transcriptional Aging Signatures in White Matter Oligodendrocytes and Immune Cells of the Monkey Brain

**DOI:** 10.1111/acel.70298

**Published:** 2025-11-24

**Authors:** Ana T. Vitantonio, Christina Dimovasili, Yuchen Liu, Bingtian Ye, Jou‐Hsuan Roxie Lee, Molly Hartigan, Benjamin Bouchard, Madelyn Ray, Bryce Conner, Kelli L. Vaughan, Julie A. Mattison, Tara L. Moore, Chao Zhang, Douglas L. Rosene

**Affiliations:** ^1^ Department of Pharmacology, Physiology & Biophysics Boston University Chobanian and Avedisian School of Medicine Boston Massachusetts USA; ^2^ Department of Anatomy & Neurobiology Boston University Chobanian and Avedisian School of Medicine Boston Massachusetts USA; ^3^ Department of Medicine Boston University Chobanian and Avedisian School of Medicine Boston Massachusetts USA; ^4^ Translational Gerontology Branch, National Institute on Aging, National Institutes of Health Baltimore Maryland USA; ^5^ Center for Systems Neuroscience Boston University Boston Massachusetts USA

**Keywords:** aging, caloric restriction, *Macaca mulatta*, microglia, myelin sheath, oligodendroglia, single nuclei RNAseq, white matter

## Abstract

During brain aging, terminally differentiated neuroglia exhibit metabolic dysfunction and increased oxidative damage, compromising their function. These cellular and molecular alterations impair their ability to maintain myelin sheath integrity, contributing to age‐related white matter degradation. Calorie restriction (CR) is a well‐established intervention that can slow biological aging and may reduce age‐related metabolic alterations, thereby preserving the molecular function of aging glia. Here we present a single nucleus resolution, transcriptomics dataset evaluating the molecular profile of oligodendrocytes and microglia in the brain of aging rhesus monkeys following lifelong, 30% calorie restriction. Oligodendrocytes from CR subjects exhibited increased expression of myelin‐related genes and showed enrichment in glycolytic and fatty acid biosynthetic pathways. In CR subjects, a subpopulation of oligodendrocytes upregulated cell adhesion gene, *NLGN1* and were in closer proximity to axons. Microglia from CR subjects upregulated amino acid and peptide metabolism pathways and showed a reduced myelin debris signature. Our findings reveal cell‐type specific transcriptional reprogramming in response to long term CR and highlight potential protective mechanisms against myelin pathology in the aging primate brain.

## Graphical abstract

Our single nuclei RNA sequencing data reveal that long‐term calorie restriction in aged rhesus monkeys reduces neuroinflammation and preserves glial metabolism. In oligodendrocytes, calorie restriction attenuates an immune signature and promotes axonal proximity, while reducing a myelin‐laden microglia population. These findings highlight calorie restriction's potential to maintain glial health relevant to myelination during aging.

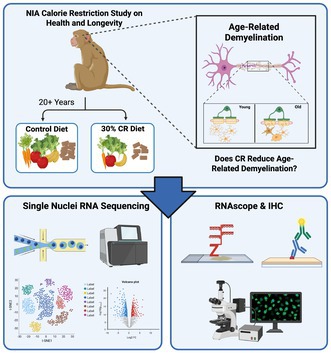

## 1 Introduction

In both monkeys and humans, normal aging is associated with subcortical white matter loss, particularly in the frontal lobe (Brickman et al. 2006; Coelho et al. 2021; Fujita et al. 2023; Guttmann et al. 1998; Marner et al. 2003; Wisco et al. 2008). This loss is accompanied by myelin pathology characterized by myelin splitting, ballooning, and redundant sheath formation, all of which disrupt normal axon conduction and correlate with cognitive impairment in aging rhesus monkeys (Bowley et al. 2010; Chen et al. 2021; Wang et al. 2020). Additional changes include increased microglia activation and infiltration of peripheral T cells into frontal white matter, both of which are correlated with cognitive impairment. However, the mechanisms underlying these changes remain unclear (Batterman et al. 2021; Bowley et al. 2010; Dimovasili et al. 2023; Shobin et al. 2017).

The rhesus monkey is a valuable model to investigate white matter aging due to its lack of neurodegenerative pathology and a similar spatiotemporal cognitive aging profile to that of humans (Dyke et al. 1986; Herndon et al. 1997; Peters et al. 1998; Wisco et al. 2008). The highly developed white matter in the monkey brain is more similar to humans than rodents, providing translational relevance (Krafft et al. 2012). Once considered purely supportive, glia are now recognized as central regulators of white matter integrity and myelin homeostasis, with their dysregulation contributing to cognitive decline (Hagemeyer et al. 2017; Hughes and Appel 2020; Mercier et al. 2024; Safaiyan et al. 2016). Transcriptomic studies have revealed that glia in frontal white matter exhibit region specific gene expression profiles, highlighting a potential unique vulnerability of white matter during aging (Hahn et al. 2023; Safaiyan et al. 2021; Wang et al. 2025). Among these glial cell types, oligodendrocytes and microglia play an integral role in maintaining myelin integrity.

Oligodendrocytes (OLs), traditionally viewed as passive myelin producers, are now recognized as active participants in dynamic cellular functions such as immune modulation and metabolic support of neurons (Chamberlain et al. 2021; Falcao et al. 2018; Jäkel et al. 2019; Looser et al. 2024; Pan et al. 2020). Aging OLs are particularly vulnerable to oxidative damage and metabolic dysfunction, likely due to the substantial energetic demands to synthesize lipid‐rich myelin, coupled with poor antioxidant defense mechanisms (Al‐Mashhadi et al. 2015; Giacci et al. 2018; Tse and Herrup 2017). These factors may ultimately drive OLs into a chronically stressed state that hinders their normal function and impacts myelination (Caprariello et al. 2015; Sim et al. 2002). Meanwhile, microglia, the innate immune cell of the brain, also play vital roles in myelin homeostasis by phagocytosing apoptotic OLs and clearing myelin debris to allow for effective remyelination (Lampron et al. 2015; Theriault and Rivest 2016). However, with age, microglia undergo morphological, transcriptional, and functional alterations that diminish their capacity to effectively phagocytose debris while perpetuating inflammatory signaling (Hammond et al. 2019; Peruzzotti‐Jametti et al. 2024; Shobin et al. 2017; Stojiljkovic et al. 2024). These age‐related dysfunctions lead to myelin pathology and contribute to reduced axon conduction, resulting in cognitive impairment (Bartzokis 2004; Dimovasili et al. 2023; Peters and Sethares 2002). Previous work from our lab has shown that white matter aging in the monkey brain is accompanied by increased myelin damage, oligodendrocyte dysfunction, and microglia inflammation, all of which are correlated with worse cognitive performance (Bowley et al. 2010; DeVries et al. 2024; Dimovasili et al. 2023; Shobin et al. 2017).

Interventions that can decelerate the accumulation of aging glial signatures and resultant myelin pathology could protect against brain aging and improve cognitive outcomes. Calorie restriction (CR) is a well‐established intervention that can slow the biological process of aging through improved energy metabolism, reduced mitochondrial dysfunction, and improved molecular waste disposal mechanisms (Mattson and Arumugam 2018; Rachakatla and Kalashikam 2022). Although longitudinal studies investigating the longevity effects of CR in rhesus monkeys have reported mixed findings (Colman et al. 2009; Mattison et al. 2017; Mattison et al. 2012), evidence from these cohorts indicate that long‐term CR may reduce oxidative OL DNA damage, attenuate reactive microglia phenotypes, and ameliorate white matter microstructural alterations (Bendlin et al. 2011; Mattison et al. 2012; Vitantonio et al. 2024). Thus, we wanted to investigate whether CR alters glial transcriptional profiles relevant to myelin pathology in the brains of old monkeys. This work was done in collaboration with the National Institute on Aging (NIA) Intramural Research Program, one of only two long term studies of CR in the rhesus monkey (Colman et al. 2009; Mattison et al. 2017). Animals from the NIA study were subjected to either a control or a 30% reduced calorie diet for over 20 years. Using single nuclei RNA sequencing and validating our findings using in situ hybridization and immunohistochemistry, we found that CR yields modest, yet significant changes in white matter glia transcriptional profiles, reducing inflammatory signatures, and promoting homeostatic functions in OLs and microglia of the monkey brain.

## 2 Results

### 2.1 Calorie Restriction Alters Glial Gene Expression While Cell Composition Remains Unchanged

**FIGURE 1:**
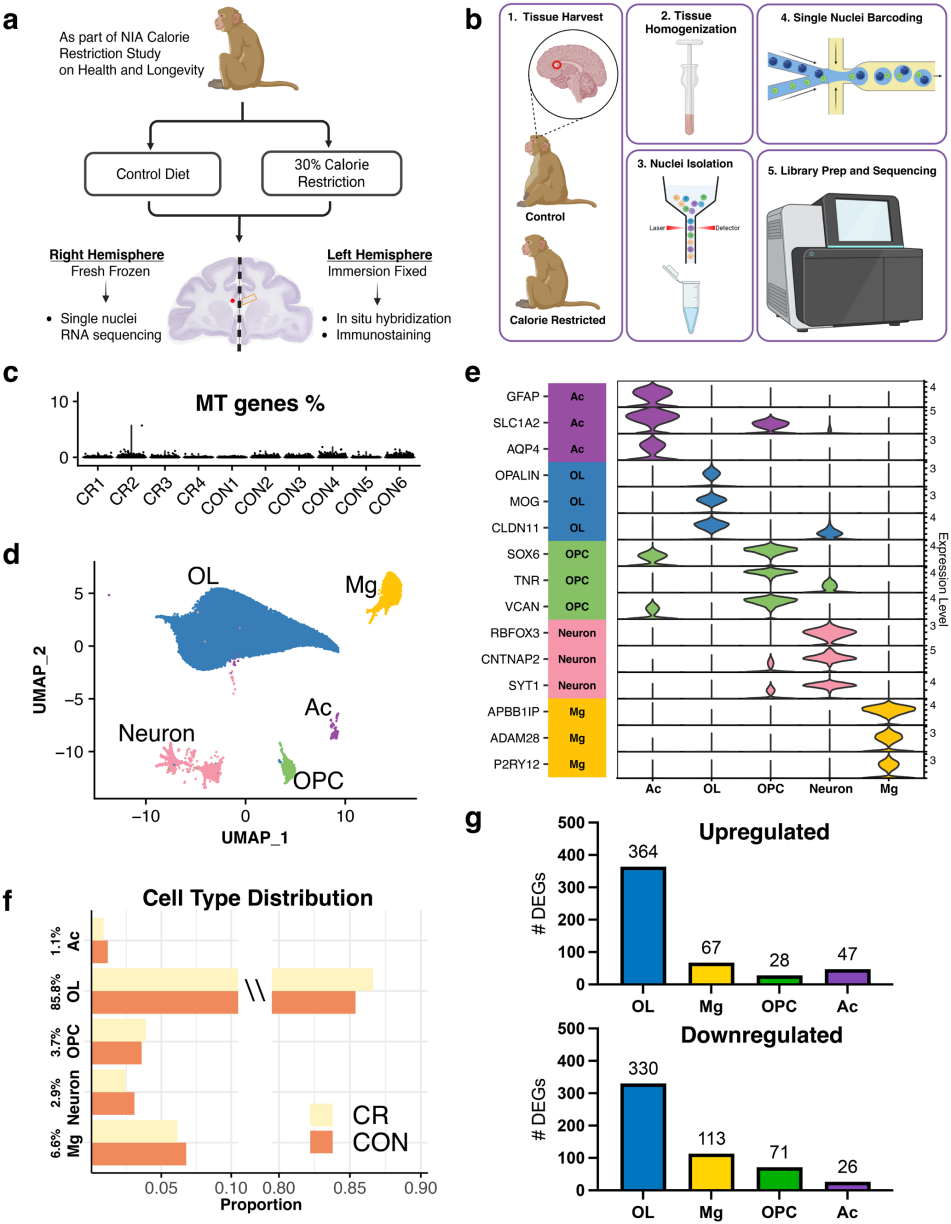
Cell type identification in the rhesus monkey corpus callosum following lifelong calorie restriction. (a) Study design and identification of region of interest. (b) Single nuclei RNA sequencing workflow using 10× Genomics platform. (c) Mitochondrial gene percentage per sample as a metric of library quality assessment (*n* = 10). (d) Uniform Manifold Approximation and Projection (UMAP) of major cell types. (e) Expression of representative cell markers per cell type. (f) Cell type distribution between control (dark orange) and CR (yellow). (g) Number of differentially expressed genes (DEG) by cell type between control and CR groups (avg(pct) ≥ 0.25, abs(log2FC) ≥ 0.25, and *p*‐value < 0.05).

White matter residing glia show dramatic transcriptional alterations with age and acquire distinct gene profiles relative to gray matter (Chen et al. 2024; Safaiyan et al. 2021), thus we wanted to investigate whether long term 30% CR could mitigate transcriptomic changes associated with myelin pathology in OLs and microglia (Figure 1a). To do this, we performed single nuclei RNA sequencing on white matter samples (approximately 30 mg) from 10 aged, male monkeys (mean age control: 33.7 years, CR: 31.5 years; 6 control, 4 CR). Tissue was collected from the anterior corpus callosum and libraries were prepared using the 10× Genomics platform (Figure 1b). Comprehensive quality control was applied to all samples. All samples showed consistently low levels of mitochondrial gene proportion, indicating high‐quality transcriptional data (Figure 1c). Cells were clustered into 5 major cell types using Seurat (Figure 1d,e). Major cell types identified were oligodendrocytes (OLs) (*OPALIN*, *MOG*, *CLDN11*), OL precursor cells (OPCs) (*SOX6*, *TNR*, *VCAN*), microglia (*APBB1IP*, *ADAM28*, *P2RY12*), astrocytes (*GFAP*, *SLC1A2*, *AQP4*), and neurons (*RBFOX3*, *CNTNAP2*, *SYT1*) (Figure 1e). OLs represent the majority of cells isolated (approx. 85%) followed by microglia (approx. 7%), with OPCs, neurons, and astrocytes in total representing < 7% of all cells captured (Figure 1f). The neuronal population isolated was likely an artifact of tissue punch location capturing deep cortical layer 6 or the white matter neurons that reside just below layer 6 (Mortazavi et al. 2017). Since this cell type was not represented across all samples, it was removed from further analyses. While some studies have reported alterations in glial numbers during aging (Fabricius et al. 2013; Hill et al. 2018; Pelvig et al. 2008; Peters 2009), cell type composition was unchanged between control and CR groups (Figure 1f). In contrast, differential gene expression analysis between CR and control revealed 1046 differentially expressed genes (DEG) across all glial cell types. The most DEGs were captured among OLs followed by microglia (Figure 1g). These data highlight that while glial cell composition remains stable, long‐term CR appears to alter gene expression. We next sought to evaluate these changes in OLs first, given their central role in maintaining myelination during aging.

### 2.2 CR Supports Homeostatic Oligodendrocyte Transcriptional Profile

**FIGURE 2:**
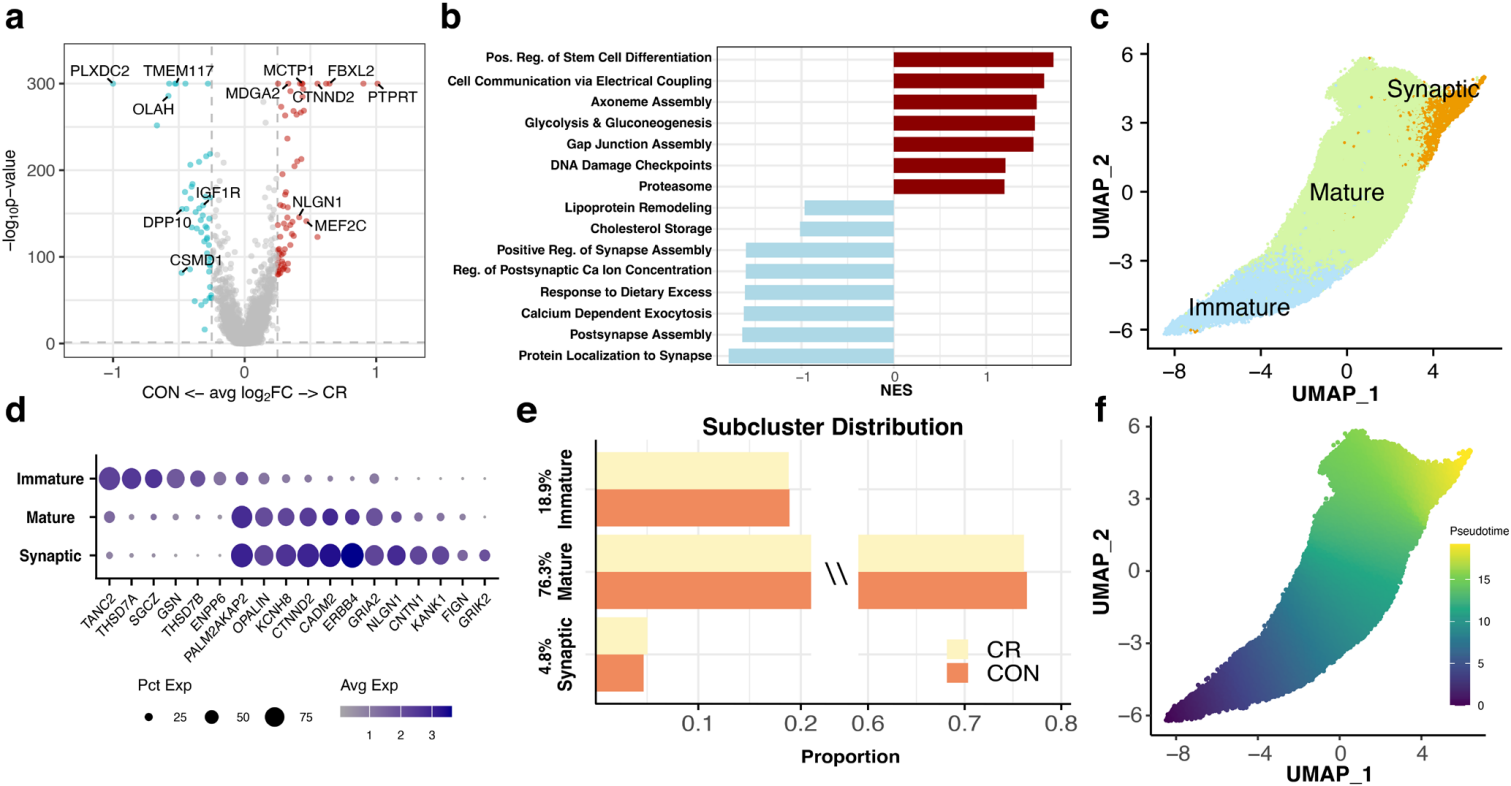
CR Maintains homeostatic transcriptional profile in oligodendrocytes. (a) Volcano plot of DEGs between OLs from control versus CR. (red = upregulated in CR, blue = upregulated in control) (avg(pct) ≥ 0.25, abs(log2FC) ≥ 0.25, and *p*‐value < 0.05). (b) Functional enrichment analysis for OL between control and CR groups. (Red = enriched in CR, blue = enriched in control). (c) UMAP of OL subclusters: Immature, mature and synaptic. (d) Top markers per subcluster. (e) OL subcluster distribution and percentage between control (dark orange) and CR (yellow). (f) Pseudotime trajectory analysis across OL subclusters.

Previous work from these same subjects showed that CR reduced mitochondrial, oxidative DNA damage in OLs, suggesting that energy utilization may be improved in response to CR (Vitantonio et al. 2024). To further assess if OLs show transcriptional alterations associated with improved energy metabolism in response to CR, we analyzed their gene expression changes and functional enrichment. We captured 694 DEG between groups (364 CR upregulated, 330 CR downregulated) with small fold changes but consistent expression patterns across cells within groups, suggesting mild yet consistent differences between groups (Figure 2a). While the magnitude of these changes is small, they are consistent with other studies of multi‐year long CR in peripheral, human tissues (Spadaro et al. 2022; Yang et al. 2016).

Functional pathway enrichment was performed to determine which biological pathways were most changed (Figure 2b). OLs from CR animals showed upregulation in stem cell differentiation, glycolysis and gluconeogenesis, electrical coupling, and gap junction formation. CR OLs also showed enrichment in some homeostatic cellular pathways like DNA damage checkpoints and proteasomal function. Additionally, top upregulated genes in CR OLs were associated with mitochondrial metabolism (*ELK3*, *AGK*), ubiquitin ligation involved in protein turnover (*ABTB2*, *KLHL28*), and cell adhesion (*CTNND2*, *NLGN1* , *MDGA2*) (Figure S1a–g). Alternatively, control OLs showed enrichment in pathways including synapse assembly & protein localization to synapses, calcium signaling, lipoprotein remodeling, and cholesterol storage. These data suggest that control OLs may shift their transcriptional profile to enhance myelin production and remodeling of the sheath while CR OLs appear to maintain a homeostatic profile, enriched in cellular maintenance pathways.

To obtain a more granular evaluation of OL transcriptomic changes, we further subclustered these cells into distinct populations. This yielded three subclusters: immature (19%), mature (76%), and synaptic (5%) OLs (Figure 2c–e). Immature OLs expressed markers consistent with OL development and some late OPC genes (*TANC2*, *THSD7A*, *SGCZ*, *ENPP6*) while mature OLs expressed markers of differentiated OLs and genes associated with myelination (*CADM2*, *OPALIN*) (Figure 2d). The final subcluster of OLs we termed “synaptic” OLs, as they were enriched for cell adhesion and glutamatergic synapse related genes ( *NLGN1* , *GRIK2*, *CNTN1*, *GRIA2*, *FIGN*) (Figure 2d), which are characteristic of OLs at the axo‐myelinic synapse as described by Stys et al. (Micu et al. 2017; Micu et al. 2016; Stys 2011). As shown in Figure 2e, CR had no effect on the distribution or the cell proportions of these subclusters (Figure 2e). Pseudotime analysis was performed to evaluate the progression of these clusters across a differentiation trajectory. This revealed cell transition from immature OLs to mature OLs, with a small subset transitioning from mature to synaptic, likely a distinct population of highly specialized, mature, myelinating OLs (Figure 2f). Since synaptic OLs showed the highest pseudotime values and may have specialized functions related to myelination, we assessed their expression profile further.

### 2.3 CR Synaptic OLs Are Enriched for Essential Myelin Synthesis Pathways

**FIGURE 3:**
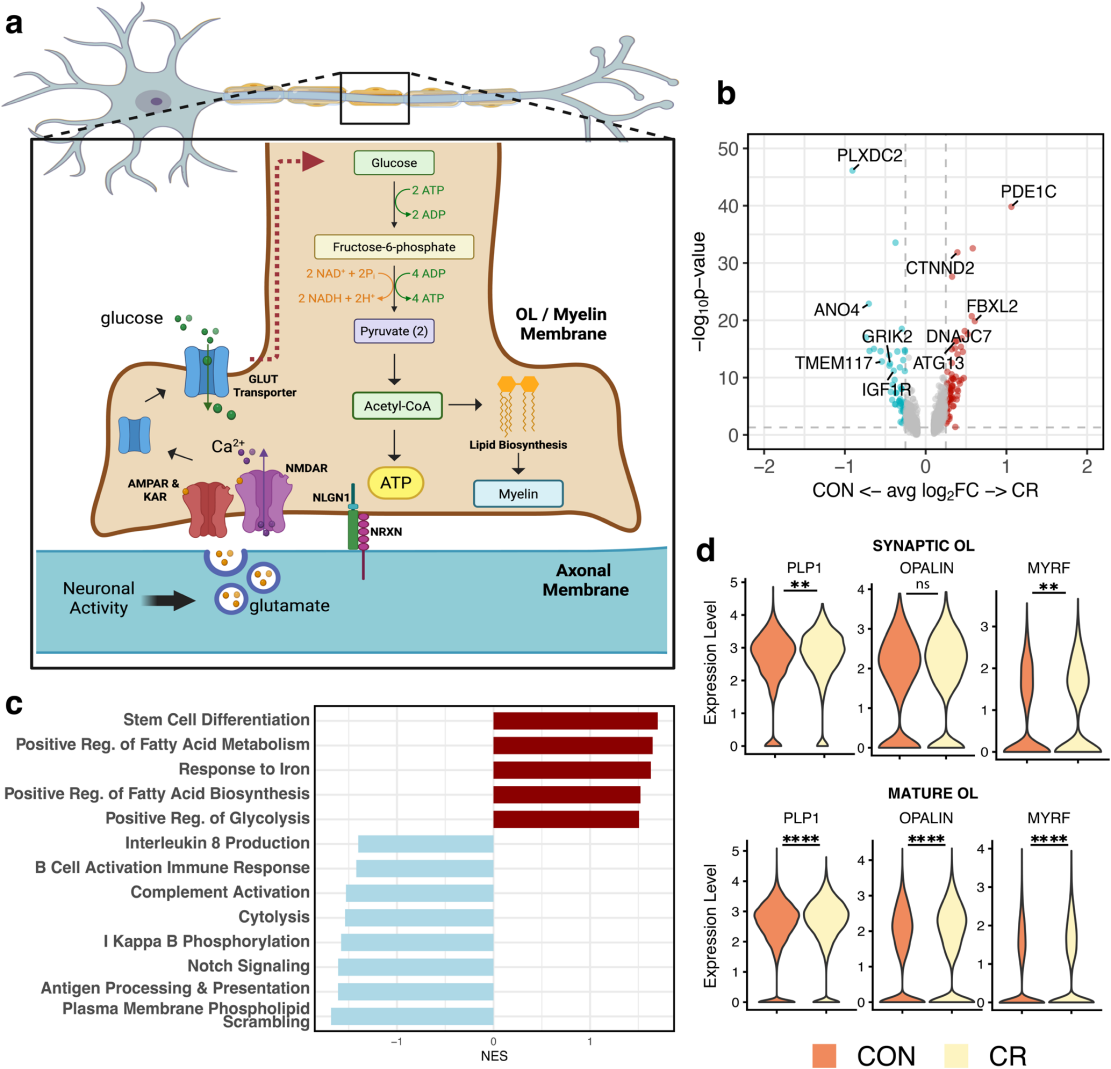
CR synaptic OL subcluster is enriched for essential myelin synthesis pathways. (a) Schematic of OL‐axon glutamatergic signaling. Activation of OL glutamatergic receptors promotes the insertion of glucose transporters into OL membrane, leading to increased glucose influx used for glycolysis to fuel lipid synthesis during myelin production (Illustration made with Biorender). (b) DEG between CR and control in synaptic OL (avg(pct) ≥ 0.25, abs(log2FC) ≥ 0.25, and *p*‐value < 0.05) (c) Functional enrichment in synaptic OL between control and CR groups. Red = enriched in CR, blue = enriched in control. (d) Expression of myelin transcripts by synaptic (top) and mature (bottom) OL between groups. (****adj‐*p* < 0.0001 ***adj‐*p* < 0.001 **adj‐*p* < 0.01).

Glutamatergic signaling from active axons activate adjacent OL glutamatergic receptors that subsequently trigger OL glucose uptake, enhancing energy influx for myelin synthesis, in an activity dependent manner (Krasnow and Attwell 2016; Looser et al. 2024; Micu et al. 2017; Micu et al. 2016; Saab et al. 2016; Stys 2011) (Figure 3a). Since synaptic OLs were enriched for glutamate receptor machinery, we examined if genes associated with this signaling pathway were altered in synaptic OLs in response to CR. We identified 408 DEG (Figure 3b) between CR and control. While both CR and control synaptic OLs expressed glutamatergic related genes, control synaptic OLs specifically upregulated expression of genes encoding glutamate receptor subunits (*GRID1*, *GRIK2*) (Figure S2a,b). *TMEM117*, a gene involved in response to ER stress and hypoglycemia was also upregulated, suggesting that control synaptic OLs may be enhancing glucose uptake via glutamate signaling (Figure S2c). Conversely, CR synaptic OLs expressed elevated transcripts involved in post synaptic organization and scaffolding (*NF1*, *DISC1*, *PICK*) and protein degradation and autophagy (*DNAJC7*, *KLHL28*, *ATG13*) (Figure 3b; Figure S2g–i). Interestingly, synaptic OL from CR animals also showed higher expression of complement inhibitors like *CD55*, suggesting immune function may be enhanced in the control group (Figure S2j). Functional enrichment confirmed an immune signature in control synaptic OLs, which showed enrichment in pathways such as antigen processing and presentation, complement system activation, interleukin 8 production, and B cell activation (Figure 3c). Alternatively, CR synaptic OLs were enriched for stem cell differentiation, positive regulation of fatty acid metabolism and biosynthesis, and positive regulation of glycolysis, all of which are essential pathways for de novo myelin synthesis (Figure 3c). To validate this, we assessed myelin gene expression and found that CR synaptic OL subclusters expressed higher levels of myelin related genes relative to controls ( *PLP1* : log2FC = 0.159, *p*‐adj = 0.004; *OPALIN* log2FC = 0.148, *p*‐adj = 0.449; *MYRF*: log2FC = 0.303, *p*‐adj = 0.0018) (Figure 3d) as did the mature subcluster ( *PLP1* : log2FC = 0.173, *p*‐adj = 1.578E‐77; *OPALIN* log2FC = 0.217, *p*‐adj = 1.682E‐73; *MYRF*: log2FC = 0.313, *p*‐adj = 9.3794E‐74) (Figure 3d). Since glutamatergic signaling across the OL‐axon interface is relevant to myelination and CR appears to differentially affect this pathway, we next sought to investigate the cell adhesion molecules that mediate this interaction.

### 2.4 *NLGN1* Is Enriched in CR Oligodendrocytes and Promotes Oligo‐Axonal Proximity

**FIGURE 4:**
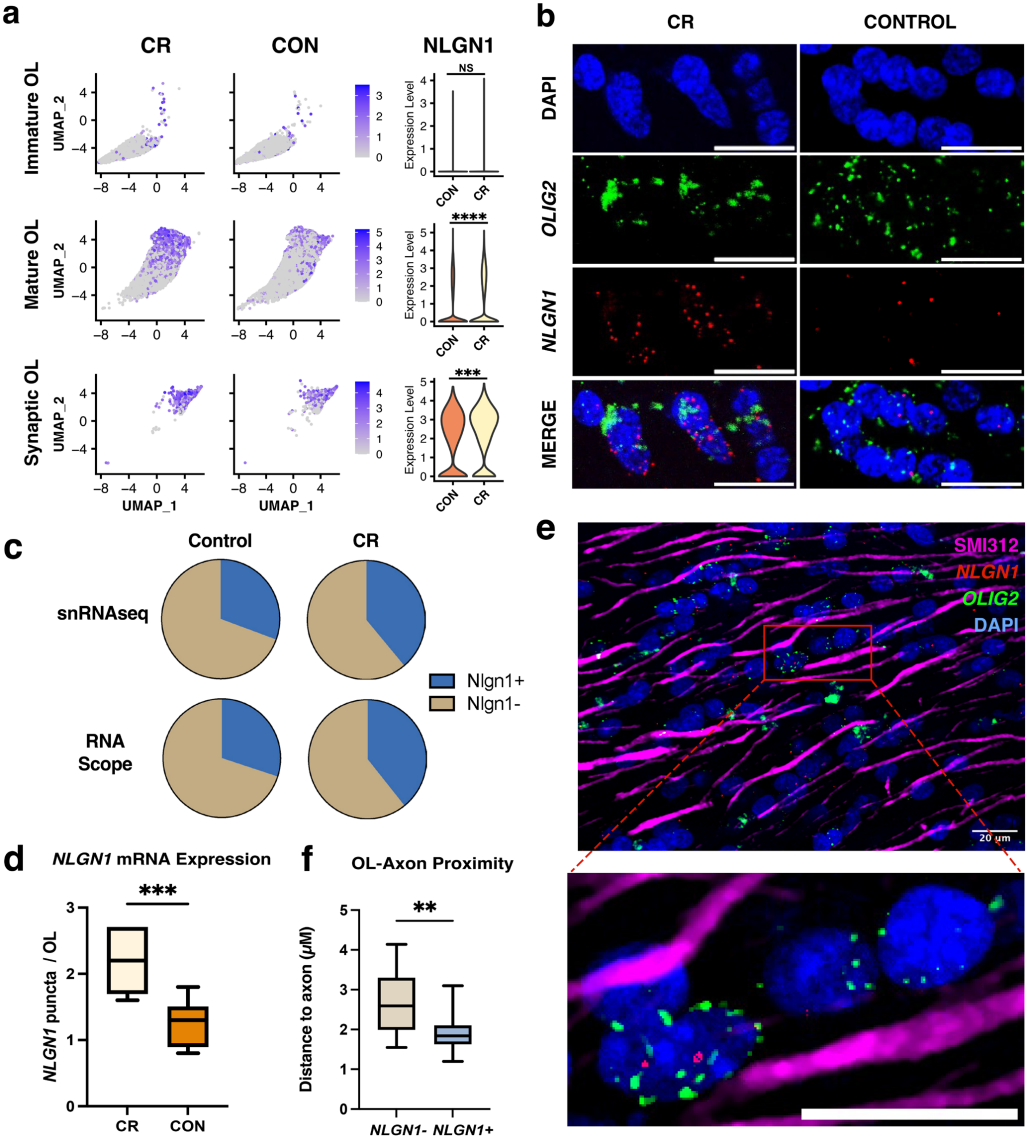
*NLGN1* is enriched in CR OL and enhances OL‐axonal contact. (a) Feature and violin plots of *NLGN1* expression across three OL subclusters between CR (left) and control (right). (b) In situ hybridization of *NLGN1* (red) within OL (*OLIG2*+ cells; green) in a CR (left) and a control (right) subject. Scale bar = 20 μm. (c) Percentage of *NLGN1* + (blue) and *NLGN1* − (tan) OL cells between control (left) and CR (right) quantified with snRNAseq (top) and with RNAScope (bottom). (d) Quantification of *NLGN1* puncta per OL nucleus per group. (e) Representative in situ hybridization combined with SMI312 immunolabeling (magenta). Scale bar = 20 μm. Inset: Magnified image. (f) Average measurements of *NLGN1* + and *NLGN1*− OL cell center distance from the nearest axon. (*N* = 14, 7 controls, 7 cal restricted; ****adj‐*p* < 0.0001 ***adj‐*p* < 0.001 **adj‐*p* < 0.01).

*NLGN1* , a post synaptic, heterophilic cell adhesion molecule that mediates the formation and maintenance of glutamatergic synapses, was highly expressed in synaptic OLs in both control and CR groups (Figure 4a). Recent studies suggest that this molecule may facilitate myelination by supporting the OL‐axon connection by tethering to a neurexin on the axonal membrane (Hughes and Appel 2019; Proctor et al. 2015). We assessed the expression of this gene and found that CR animals expressed higher levels of *NLGN1* in mature and synaptic OLs relative to controls ( *NLGN1* immature: log2FC = 0.604, *p*‐adj = 1; mature: log2FC = 0.512, *p*‐adj = 3.41E‐147; synaptic: log2FC = 0.257, *p*‐adj = 1E‐4) (Figure 4a). To validate this finding and assess if *NLGN1* expression altered OL‐axon proximity, we performed in situ hybridization on the anterior corpus callosum from the contralateral hemisphere of 14 male and female animals (7 control, 7 CR; 7 female, 7 male) (Figure 1a; Table 1). Using RNA probes for *OLIG2* and *NLGN1* , we identified OLs (*OLIG2+*) and quantified *NLGN1+ OLIG2+* cells (Figure 4b–d). We found a 30% increase in the number of *OLIG2+* cells co‐expressing *NLGN1* in the CR group relative to controls (Con: *NLGN1* + *OLIG2*+ = 30.0%; CR: *NLGN1* + *OLIG2*+ = 39.2%). This is consistent with proportions of *NLGN1* + OLs identified from snRNA sequencing (Figure 4c). Next, we quantified the amount of *NLGN1* mRNA puncta within *OLIG2*+ cells. Consistent with our snRNA seq findings, there was a 76% increase in *NLGN1* puncta in CR OLs compared to controls (*p* = 0.0008) (Figure 4d). Multiple regression revealed no effect of sex nor age on *NLGN1* mRNA (age: 0.9903; sex: 0.3211). We performed concurrent immunostaining for SMI312, a marker of axons (Figure 4e), and analyzed the proximity of *OLIG2+* cells to axons. Results showed that *NLGN1* + *OLIG2*+ cells were significantly closer to axons than *NLGN1* − *OLIG2*+ cells (CR: 1.92 μm, Con: 2.70 μm; *p* = 0.0046) (Figure 4f). Together, these data show that synaptic OLs are enriched for *NLGN1* expression, which is more highly expressed in synaptic OL from CR subjects. In situ validation confirmed that CR animals express higher levels of *NLGN1* and have more *NLGN1* + OLs, which are in closer proximity to axons than *NLGN1* − OLs. Following in situ validation of this snRNAseq finding within OLs, we next shifted our focus to the immune cells of the brain to see if gene expression and pathways enrichment changes were conserved across cell types.

**TABLE 1:** Subject demographics.

A	Animal ID	Age	Age onset	Sex	Nuc seq	RNA scope	IHC
Controls	CR025	26.7	4.7	F		X	X
	CR027	27.9	2.1	M			X
	CR056	31	1.8	F			X
	CR034	32.6	3.83	M			X
	CR060	31.5	2.8	F		X	X
	CR047	32.8	7.7	F		X	X
	CR059	33.3	2.1	M	X	X	X
	CR050	33.4	1.8	M	X	X	X
	CR039	34.1	4.8	M	X		X
	CR058	34.5	1.9	M	X	X	X
	CR044	33.8	1.75	F			X
	CR057	32.3	0.78	M	X		
	CR035	34.7	1.8	M	X	X	X
Calorie restricted	CR001	22.4	1.8	F			X
	CR020	23.5	1.8	F		X	X
	CR021	28.7	1.8	M	X	X	X
	CR033	30.7	1.8	M			X
	CR024	30.9	3.9	M	X	X	X
	CR030	31.4	1.8	F		X	X
	CR029	31.7	2.7	F		X	X
	CR042	31.9	1.8	M	X	X	X
	CR048	33	7.6	F			X
	CR053	34	6.5	F		X	X
	CR061	34.4	2	M	X		X
	CR043	36.8	1.8	M			X

*Note:* (A) Demographic details and tissue availability of animals in each experiment.

### 2.5 CR Alters Microglia Gene Expression and Reveals a Myelin‐Debris‐Associated Subtype

**FIGURE 5:**
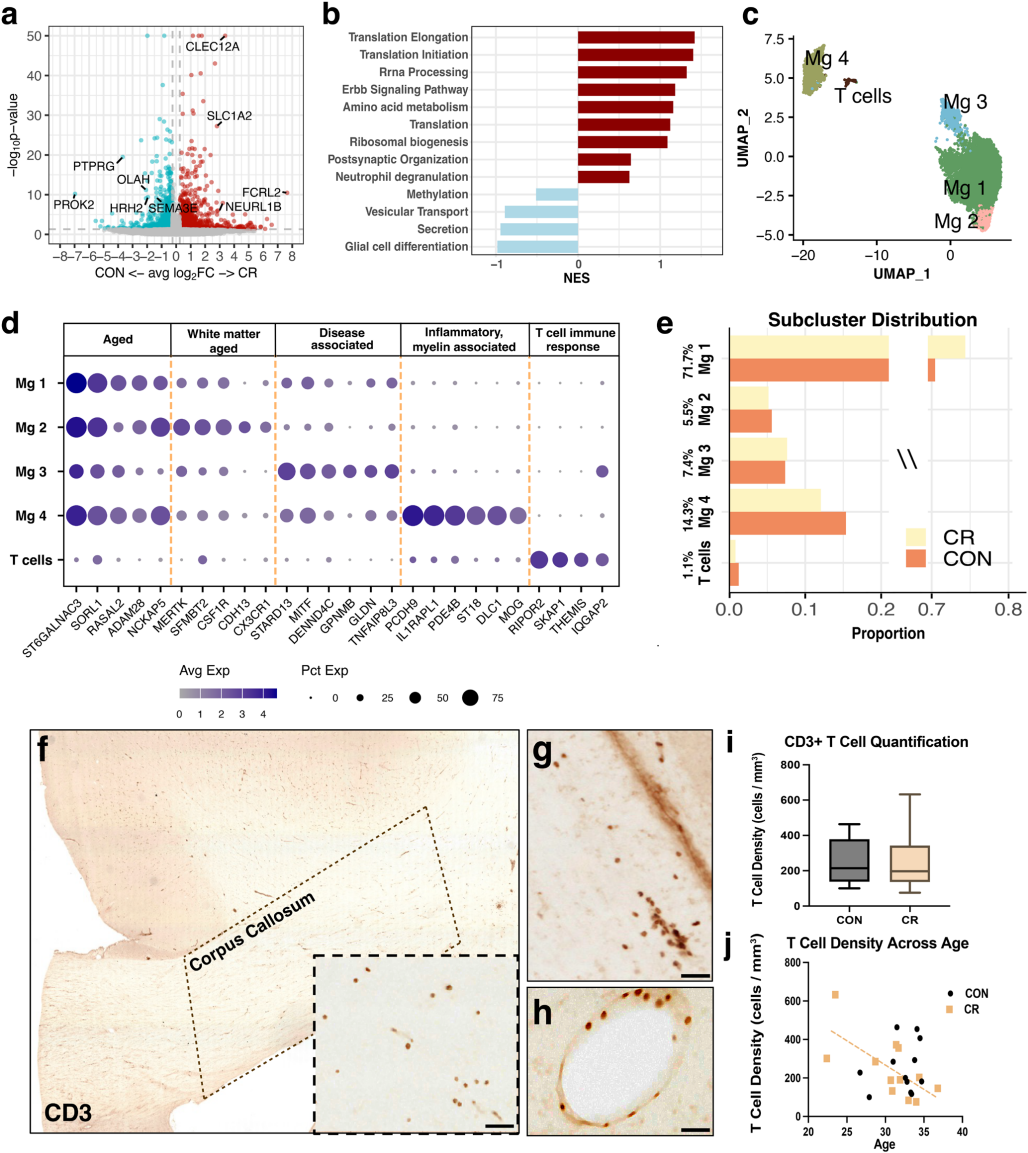
CR Microglia are enriched for protein synthesis and metabolic pathways. (a) DEGs between control and CR microglia (avg(pct) ≥ 0.25, abs(log2FC) ≥ 0.25, and *p*‐value < 0.05) (b) Functional enrichment analysis performed for all microglia between control and CR groups. Red = enriched in CR, blue = enriched in control. (c) UMAP of 4 microglia subclusters: Mg1, 2, 3, and 4 and a T cell population. (d) Top markers per subcluster. (e) Microglia subcluster distribution between control (dark orange) and CR (yellow). (F) CD3 immunohistochemistry confirms T cell infiltration into brain parenchyma in the corpus callosum. (g) T cells infiltrate in a cluster like pattern. (h) Perivascular T cells were excluded from all quantifications. (i) Quantification of CD3^+^ T cell density in the corpus callosum of CR and control subjects. (j) Linear regression of T cell density across age between CR (light orange) and control (black) (*N* = 24). (f–h) scale bar = 50 μm.

We first assessed differential gene expression between control and CR microglia, identifying 180 significantly altered genes (67 upregulated, 113 downregulated in CR) (Figure 5a). Notably, *PRKAG3*, encoding a regulatory subunit of AMP‐ activated protein kinase (AMPK) was upregulated in CR microglia, suggesting sustained AMPK pathway activation following two decades of CR. Pathway analysis revealed upregulated translation processes in CR microglia including translation initiation and elongation, ribosome biogenesis, and rRNA processing (Figure 5b). Enrichment in amino acid and peptide metabolism further indicated enhanced protein metabolism and metabolic activity in CR microglia. In contrast, control microglia showed enrichment in stress‐related pathways including response to oxygen and nitrogen species, secretion, and vesicular transport (Figure 5b).

Subclustering identified 5 distinct microglial populations (Figure 5c). Subclusters 1–3, together, comprised about 86% of all microglia and shared similar markers of aged microglia. Subcluster 2 expressed high levels of white matter associated genes (*NRP1*, *MERTK*, and *NCKAP5*) (Figure 5d) while subcluster 3 displayed a disease‐associated signature (*STARD13*, *MITF*, *GPNMB*, and *DPYD*) previously observed in Alzheimer's disease (AD) and Multiple Sclerosis (MS) (Farzan et al. 2025; Guvenek et al. 2024; Pettas et al. 2022; Srinivasan et al. 2020; van den Bosch et al. 2024). However, subclusters 1–3 did not differ between groups or show meaningful associations with myelin pathology and thus, were not investigated further (Figure 5e).

Microglia subcluster 4 (14.3%) showed a unique expression profile enriched with myelin and OL‐specific genes (*PCDH9*, *Il1RAPL1*, *ST18*, *MOG*, and *PDE4B*) (Figure 5d), consistent with prior reports of OL and myelin transcripts accumulation in the nuclear compartment of actively phagocytosing microglia (Chen et al. 2024; Schirmer et al. 2019). Subcluster 5 (1.1%) co‐clustered with microglia but uniquely expressed T cell‐specific genes and CNS homing markers *ITGAL*, *ITGA4* (Glatigny et al. 2015; Smolders et al. 2018), while lacking *CCR7* expression, a marker of naïve, peripheral T Cells (Larbi and Fulop 2014) (Figure S3a–c) suggesting these are CNS‐infiltrating T cells rather microglia with a T cell signature. Both this population and disease‐associated microglia (subcluster 3) expressed *IQGAP2*, a gene that has been involved in the regulation of immune cell traffic across the BBB (Katdare et al. 2025) (Figure S3d).

To validate the T cell infiltration and assess if CR modulates T cell abundance, we stained brain tissue from 24 male and female monkeys (12 control, 12 CR; 11 female, 13 male) for CD3^+^ T cells (Figure 5f). T cells were observed in a cluster‐like pattern in the brain parenchyma (Figure 5g). Perivascular T cells were excluded from our analyses as we wanted to quantify infiltrated T cells (Figure 5h). No significant differences in parenchymal T cell density were observed between groups (*p* = 0.4189) (Figure 5i), suggesting CR does not directly alter T cells infiltration. Multiple linear regression revealed no sex‐specific alterations in T cell infiltration (*p* = 0.3625), though CR reduced the rate of T cell accumulation with age in the brain (*p* = 0.0444; Con: *R* ^2^ = 0.057, CR: *R* ^2^ = 0.463) (Figure 5j).

Given its unique myelin enriched signature and potential relevance to OL function, we next investigated microglia subcluster 4 for its involvement in myelin pathology.

### 2.6 Microglia With Myelin Debris Signature Are Less Abundant in CR

**FIGURE 6:**
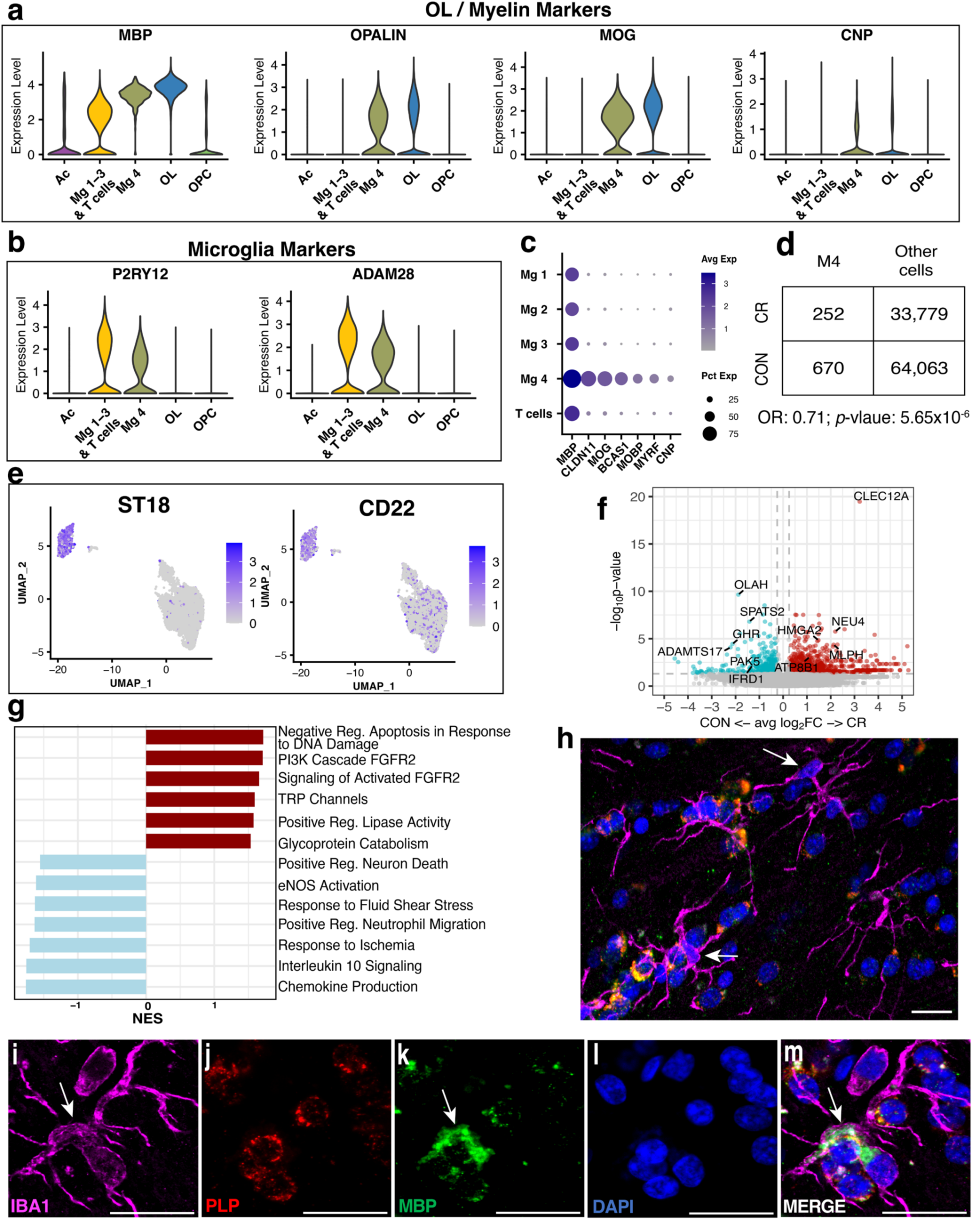
Microglia with myelin debris signature are less abundant in CR. (a) Microglia 4 express OL and myelin related genes (*MBP*, *OPALIN*, *MOG*, *CNP*) (b) as well as typical microglia markers (*P2RY12* and *ADAM28*). (c) OL and myelin genes are uniquely expressed by microglia 4 among other microglia subclusters. (d) Controls show a larger representation of Microglia 4. (e) Microglia 4 are enriched for myelin debris processing genes, *ST18* and *CD22*. (f) Volcano plot of DEG between control and CR microglia 4 (avg(pct) ≥ 0.25, abs(log2FC) ≥ 0.25, and *p*‐value < 0.05). (g) Functional enrichment analysis for microglia 4 in the CR group. (h–m) In situ hybridization of *PLP1* (red) and *MBP* (green) mRNA in IBA1+ (magenta) microglia shows *MBP* inclusions localize in the perinuclear compartment, rather than within the nucleus (blue) of microglia. (h) scale bar = 10 μm. (i–m) scale bar = 20 μm.

Microglia subcluster 4 uniquely expressed both OL and myelin specific genes and canonical microglia markers (Figure 6a–c). Indeed, this population showed elevated expression of lipid transport and metabolism genes ( *ABCA2* , *ABCA8* , *SCARB1* , *SCARB2* ), supporting a role in myelin debris uptake (Figure S4a–d). We observed an approximate 29% reduction in the total proportion of microglia 4 relative to all cells captured in CR compared to control animals (Figure 6d). Notably, this population was enriched for *ST18*, which is associated with active myelin engulfment (Schirmer et al. 2019), and *CD22*, a negative regulator of myelin phagocytosis, implying a hindered myelin phagocytic process (Figure 6e). Interestingly, top DEGs in CR were associated with regulating inflammation and mitigating oxidative stress (*CD5L*, *ATP5F1A*, *MLPH*). Functional pathway analysis revealed enrichment for activated FGFR2 and FGFR2/PI3K signaling, pathways of which are implicated in cell survival, and other metabolic pathways (Figure 6g). Alternatively, control microglia 4 showed a more classic, inflammatory profile with enrichment for chemokine production, interleukin 10 production, and neutrophil migration (Figure 6g).

Other studies using single nuclei sequencing, including human studies of multiple sclerosis, report copurification of myelin transcripts within actively phagocytosing microglia (Li et al. 2019; Schirmer et al. 2019; Solga et al. 2015). These transcripts have been shown to localize to both the nuclear and perinuclear compartment of these cells. To validate our results and visualize the expression and spatial localization of myelin transcripts within microglia, we used in situ hybridization to assess *MBP* and *PLP1* mRNA within IBA1+ microglia (Figure 6h–m). *MBP* and *PLP1* mRNA was not observed in the nuclear compartment (Figure 6h), although perinuclear *MBP* inclusions were detected (Figure 6i–m). These data support that this subpopulation of microglia demonstrate a gene signature consistent with myelin debris engulfment and potentially arrested phagocytosis. CR microglia 4 upregulated anti‐inflammatory genes while controls showed enrichment in inflammatory pathways. As observed in synaptic OLs, CR likely dampens immune signaling in microglia 4.

## 3 Discussion

Age‐related changes in OLs and microglia disrupt myelin homeostasis, contributing to myelin degradation and neuroinflammation. While CR is known to slow biological aging across multiple species, its effects on brain aging in higher organisms are not clear. This study demonstrates that CR can modulate aging glial biology by preserving metabolic function and reducing inflammatory signatures in the monkey brain. In OLs, CR maintained glucose metabolism and myelin gene expression, supporting the oligo‐axon connection while suppressing immune‐like responses. In microglia, CR decreased the abundance of myelin‐laden cells and promoted a more anti‐inflammatory state.

### 3.1 Calorie Restriction Supports Oligodendrocyte Metabolism

Overall, the composition of glia isolated from the anterior corpus callosum did not differ between CR and control conditions, though gene expression analysis and functional enrichment identified modest yet significant changes, demonstrating the ability of long‐term CR to alter aging glial biology. OLs were the most abundant cell type captured in our samples of white matter and showed the largest response to CR. OLs are selectively vulnerable to metabolic dysfunction and show reduced glycolytic capacity and altered mitochondrial function in response to aging (Fünfschilling et al. 2012; Zhang et al. 2020). This metabolic dysfunction impairs myelination capacity and drives oxidative damage that compromises myelin sheath integrity (Asadollahi et al. 2024; Bartzokis 2004). Here we found that genes involved in mitochondrial metabolism and glycolytic and gluconeogenic pathways were enriched in CR OLs, supporting the hypothesis that CR combats age‐associated metabolic decline. Additionally, cellular communication and gap junction formation pathways were enriched in CR OLs. OLs form gap junctions with surrounding astrocytes as a means to coordinate neural activity and transport metabolites like glucose, of which, are reduced in post mortem MS lesions. This suggests that CR may maintain metabolic coupling between glia (Hu et al. 2023; Markoullis et al. 2014; Wasseff and Scherer 2011). Alternatively, control OLs showed enrichment in lipoprotein remodeling, cholesterol storage, and synapse formation. While these pathways support myelin synthesis and formation, their enrichment may reflect a compensatory response to myelin damage that requires remyelination.

### 3.2 Calorie Restriction Supports Synaptic Adaptations and Metabolic Coupling in Oligodendrocytes

Upon more thorough analysis of the OL cell type, we identified three OL subclusters: immature, mature, and synaptic. While some studies report a reduction in mature, myelinating OLs with age, we found no effect of CR on the total number or subcluster distribution of OLs (Fabricius et al. 2013; Pelvig et al. 2008; Soreq et al. 2017). Synaptic OLs, the smallest but most specialized OL subcluster, as a whole, showed enhancement in glutamatergic signaling, cell adhesion, and myelin gene expression, reinforcing this subcluster's implication in myelination. While the molecular mechanisms of OL‐axon communication via synaptic‐like processes are not fully established, recent studies have characterized the physiology of the “axo‐myelinic synapse,” a non‐canonical, chemical synapse that utilizes glutamatergic signaling to modulate myelination and metabolic support in an activity dependent manner (Micu et al. 2017; Micu et al. 2016; Stys 2011). Synaptic vesicle release at specific axonal regions has been shown to promote myelination, implicating glutamatergic signaling in myelin regulation (Almeida et al. 2021). More specifically Saab et al. (2016) showed that activation of OL glutamatergic receptors promotes glucose uptake through insertion of GLUT1 transporters into the myelin membrane in a calcium dependent manner (Krasnow and Attwell 2016; Micu et al. 2017; Saab et al. 2016). In our study, control synaptic OLs exhibited downregulated glycolytic and fatty acid biosynthetic pathways and upregulated expression of glutamate receptor genes, potentially as a compensatory response to promote glucose influx required for myelin synthesis. Alternatively, CR synaptic OLs were enriched for glycolysis and fatty acid biosynthesis and post synaptic organization, suggesting improved glucose handling and metabolic coupling, potentially an adaptation to reduced glucose availability across the lifespan. Together, these findings highlight a potential divergence in how synaptic OLs utilize glutamatergic signaling during aging where CR synaptic OLs are orientated towards sustaining the OL‐axon connection while control synaptic OLs exhibit a compensatory but potentially maladaptive response to metabolic stress.

Other studies report the expression of synaptic proteins, including NLGN1, by OLs and demonstrate that this expression modulates myelin sheath formation (Hughes and Appel 2019). *NLGN1* is enriched at glutamatergic synapses, which are crucial during myelination, particularly activity dependent myelination and remyelination, where glutamate can stimulate local *MBP* translation, support metabolic coupling, and promote OPC maturation (Gautier et al. 2015; Turan et al. 2021). *NLGN1* expression was elevated in CR synaptic OLs with a larger proportion of *NLGN1* expressing OLs and higher levels of *NLGN1* mRNA. This upregulation is likely related to the elevated myelin gene expression we also observed synaptic CR OLs. We additionally found that *NLGN1* expressing OLs were in closer proximity to axons than *NLGN1* negative OLs, possibly facilitating or strengthening the OL‐axon connection.

### 3.3 Calorie Restriction Mitigates Age‐Related Immune Alterations

It is also important to note that synaptic OLs in controls showed enrichment in many immune pathways when compared to the CR group. Previous reports of immune‐oligodendrocytes in the literature support a role in orchestrating inflammatory responses under neuropathological conditions, including antigen presentation to peripheral immune cells (Falcao et al. 2018; Jäkel et al. 2019; Kirby et al. 2019). Enrichment in immune pathways within control synaptic OLs may be triggered by abnormal Ca^2+^ levels or accumulating myelin debris, both of which may be less abundant in the CR condition (Alberdi et al. 2002). The adoption of immune like functions within control synaptic OLs may also influence local microglia dynamics, potentially promoting a reactive state or interfering with myelin debris clearance.

Similar to OLs, CR enhanced aspects of microglia metabolism including upregulation of amino acid metabolism and protein synthesis pathways. We identified five subclusters of microglia, including microglia 4, which was enriched in myelin‐related transcripts. RNAscope confirmed *MBP* mRNA inclusions within the perinuclear space, consistent with internalized transcripts following myelin phagocytosis. A similar population were described in human MS patients by Schirmer et al. (Schirmer et al. 2019) who showed that *mbp* and *plp* transcripts persist in microglia up to 4 days following ingestion, suggesting potential recent myelin uptake by microglia 4 prior to nuclei isolation. Consistent with Schirmer's findings, microglia 4 expressed lipid transport and metabolism genes and was enriched for *CD22* expression, a negative regulator of microglia phagocytosis that are upregulated with age (Aires et al. 2021). Blockade of CD22 in microglia has been shown to restore homeostatic phagocytosis in the aging brain, indicating that microglia 4 may be myelin‐laden with ineffective phagocytic capacity (Pluvinage et al. 2019; Ren et al. 2023). CR reduced microglia 4 abundance, suggesting CR may preserve phagocytic function or limit myelin degeneration in the first place. Similar to synaptic OLs, CR prevented enrichment in immune signaling pathways in these microglia, suggesting that reduced inflammation mediated by CR is not cell type specific, but potentially a global change in the brain environment. While we have shown CR dampens glial immune signaling, age‐related neuroinflammation is also driven by infiltrated peripheral immune cells, which we have shown increase with age and are correlated with cognitive decline in the aging monkey brain (Batterman et al. 2021). While CR did not reduce total T cell numbers, it slowed age‐related T cell accumulation, possibly reflecting improved health span, or successful aging.

### 3.4 Limitations and Future Directions

A limitation of this study is that our sequencing data was only performed in males. While we attempted to offset this by performing validation in both males and females, our sample size is low and thus, the effect of sex as a biological variable still warrants further investigation. In addition, our findings provide proof‐of‐concept support for calorie restriction‐mediated molecular relationships. Given the limitations of post‐mortem brain tissue, future studies will utilize mechanistic approaches to clarify the precise cellular and molecular pathways mediating our observations.

## 4 Conclusion

In summary, we found long‐term calorie restriction (CR) to broadly reduce neuroinflammation and preserve metabolic function across multiple glial cell types. We provide novel evidence that lifelong CR in rhesus monkeys modulates oligodendrocyte (OL) glycolytic and fatty acid biosynthetic pathways relevant to myelination. We identified a unique OL subtype implicated in myelination and enriched for glutamatergic signaling, in which CR attenuated an immune response and promoted axonal proximity. Additionally, we show that CR reduces the expression of a myelin‐laden microglia population with potentially impaired phagocytic capacity. Our findings suggest that OLs and microglia are metabolically responsive to nutrient availability and that the highly developed white matter of the monkey brain offers a valuable model for studying dietary interventions. These results support CR's potential to protect glial health and myelination in aging, potentially extending brain health span during aging.

## 5 Materials and Methods

### 5.1 Subjects

For this study, a total of 24 male and female rhesus monkeys between the ages of 22 and 37 years (median = 32.1), approximately equivalent to 66–108 human years (Tigges et al. 1988), were selected based on tissue quality and availability (Figure 1a; Table 1). The subjects used in this study were part of a larger, longitudinal study conducted at the National Institute on Aging (NIA) to investigate the effects of long‐term CR on health and longevity, which is described in detail elsewhere (Ingram et al. 1990; Mattison et al. 2005; Mattison et al. 2017; Mattison et al. 2012). CR monkeys consumed about 30% fewer calories than their age‐, sex‐, and body weight‐matched controls (450–600 vs. 600–800 daily), as previously described in Mattison et al. (2012) and Ingram et al. (1990) (Figure 1b). Subjects were individually housed at the NIH Animal Center in Poolesville, MD. Individual housing was required to ensure precise measurement of food consumption for calorie restriction protocols and to prevent aggression, though animals maintained visual, auditory, and olfactory contact with other monkeys in accordance with IACUC‐ approved standards. Monkeys were provided with a specially formulated diet (NIA‐1‐87) that included a 40% surplus of vitamins and minerals to compensate for reduced intake in CR monkeys. Diet composition is reported in Ingram et al. 1990. Monkeys were euthanized when clinically indicated, at which time brains were collected with one hemisphere cut into slabs and fresh frozen while the other hemisphere was immersion fixed in 4% paraformaldehyde. Both types of samples were sent for processing at Boston University School of Medicine (BUSM) to investigate the effects of CR on brain aging.

### 5.2 Brain Tissue Harvest and Storage

For euthanasia, subjects were sedated with an intramuscular injection of Telazol (3–6 mg/kg) and administered a lethal intravenous dose of sodium pentobarbital (50 mg/kg). A craniotomy was performed, and the brain was removed and hemisected at the midsagittal plane (Figure 1b). The right hemisphere was sliced into 1 cm thick coronal sections, snap frozen in liquid nitrogen, and stored at −80°C, maintaining a postmortem interval of ≤ 3 h for all subjects. Previous evaluation of unfixed brain tissue from the NIH demonstrate that high quality RNA can be isolated from tissued stored at −80°C for up to 23 years (White et al. 2018). Sections were shipped to BUSM on dry ice for processing. The left hemisphere was immersed in fresh 4% paraformaldehyde (pH 7.4) at 4°C for 7 days, during which time it was shipped overnight on cold packs to BUSM. After 7 days of fixation, the hemisphere was blocked, in situ in the coronal stereotactic plane and cryoprotected sequentially in 10% glycerol, 2% DMSO, 0.1 M phosphate buffer for 2 days, followed by 20% glycerol, 2% DMSO, 0.1 M phosphate buffer for 1 week. The cryoprotected hemisphere was flash frozen in −75°C isopentane and stored at −80°C until cut using a sliding microtome into 10 equally spaced series of 30 μm thick sections. Sections were collected in cryoprotectant (15% glycerol, 0.1 M phosphate buffer) except for one series used for RNAscope, which included an additional 1% paraformaldehyde for RNA preservation. All sections were stored at −80°C until further processing. These fixed tissue preservation method preserves antigens and immunohistochemical reactivity for a minimum of 10 years (Estrada et al. 2017; Rosene et al. 1986).

### 5.3 Nuclei Isolation and Library Preparation

Single nuclei libraries were prepared from 10 male monkeys, 6 control and 4 CR, based on fresh frozen tissue availability (fresh frozen tissue from females was not yet available from a sufficient number of females). For processing, tissue was removed from the freezer and immediately placed on dry ice. A dermal punch was used to isolate a 2 mm sample from the frontal region of the corpus callosum (Figure 1d). Tissue was homogenized using a Dounce homogenizer in a nuclei isolation buffer (0.25 M sucrose, 25 mM KCl, 5 mM MgCl2, 1 mM DTT, 0.1%. Triton X, 1X protease inhibitor, and 1 μm DAPI in nuclease free water). Following homogenization, the tissue lysate was filtered through a 40 μm cell strainer on ice and samples were transferred to a 5 mL polypropylene round‐bottom FACS tube. Nuclei were sorted by fluorescence activated cell sorting (FACS) using a FACSAria sorter and DAPI fluorescence. 50,000 nuclei were sorted per sample and collected in nuclei storage buffer (0.25 M sucrose, 5 mM MgCl2, 10 mM Tris HCl, 2% BSA, and 1× protease inhibitor in nuclease free water). Nuclei concentrations were validated with trypan blue staining and counted with a hemocytometer with approximate concentrations of 400 nuclei/μL. Barcoding and library preparation was completed using the 10× Chromium Next GEM Single cell 3′ Kit (v3.1) by 10× Genomics. Samples were batch processed in balanced groups. Approximately 10,000 nuclei were loaded onto Chip G (10× Genomics), barcoded, and individual libraries were generated. Quality control at the steps of cDNA and library generation was performed using an Agilent Bioanalyzer High Sensitivity DNA chip to determine fragment size. Libraries were sequenced using the Illumina NextSeq 2000 at the BUSM microarray and sequencing core facility.

### 5.4 Single Nuclei RNA Seq Data Processing

Fastq files from single‐nucleus mRNA sequencing were aligned to the *Macaca mulatta* reference genome (Mmul10, release 105, GCA_003339765.3) using the CellRanger pipeline (version 6.1.2). The quality control (QC) report demonstrates consistent and high‐quality libraries across all samples (Table S1). Additional comprehensive QC was performed using the Seurat R package, filtering out low‐quality cells with mitochondrial or ribosomal gene content > 5%. Clustering analysis was performed on each individual sample, and major cell types were identified based on canonical markers reported in previous publications (see Figure 1e). The 10 high‐quality libraries were integrated using Seurat. No significant batch effects were observed. The integrated dataset was normalized and scaled, followed by PCA and UMAP for clustering and visualization. Marker genes were identified using Seurat's “FindMarkers” function across different conditions and cell populations and curated based on literature. The default Wilcoxon rank‐sum test and Bonferroni correction were used to identify markers between groups. Differentially expressed genes (DEGs) were defined as those expressed in more than 25% of cells with an *p*‐value < 0.05 and abs(log2FC) ≥ 0.25 between groups. Functional enrichment analysis of DEGs was performed using GSEA, GO terms, KEGG pathways, and Reactome pathways, with an enrichment *p*‐value threshold of < 0.05. For each major cell type, the above procedure was repeated to identify cell subtypes and their corresponding markers. The Monocle 3 package was then used to infer pseudotime trajectories across the different subpopulations.

### 5.5 In Situ Hybridization and Immunofluorescence

To validate snRNAseq data, RNAscope hybridization for selected genes was performed using the RNAscope Multiplex Fluorescent Manual Assay kit (323100, ACD, CA) on brain tissue from a subset of male and female monkeys (*n* = 14). 30 μm thick brain sections stored in 15% glycerol, 1% PFA, 0.1 M phosphate, were thawed from −80°C and the corpus callosum was dissected out from one frontal section per subject. Sections were treated with H_2_O_2_ for 10 min at room temperature (RT) and mounted on SuperFrost Plus slides. Target retrieval was performed for 5 min at 95°C in target retrieval buffer (ACD, CA) followed by a 30 min incubation in Protease plus (ACD, CA) at 40°C in the HybEZ oven (ACD, CA). Tissue was incubated with primary probes (*OLIG2* [1203071‐C1, ACD, CA] and NLGN1 [1572231‐C4, ACD, CA] or MBP [1006431‐C1, ACD, CA] and PLP1 [1057381‐C2, ACD, CA]) for 2 h at 40°C, then stored overnight in 5 × SSC hybridization buffer. The next day, tissue underwent probe amplification steps and fluorophore conjugation. Fluorescent probes were diluted 1:1000 in TSA buffer (ACD, CA) (*OLIG2*: Opal 520; NLGN1: Opal 570; *MBP*: Opal 520; PLP1: Opal 570) (Akoya Biosciences, MA). Positive and negative controls were included in each experiment using a custom housekeeping cocktail (#521,081, 461,341, 457,711) and negative control mix (#320,878). Immediately following RNAscope, tissue was blocked in Superblock (ThermoScientific #37515) for 1 h at 40°C in the HybEZ oven, then incubated in primary antibody against SMI312 (1:250, BioLegend, 837904) or IBA1 (1:500, Wako, 011‐27991) diluted in Superblock for 1 h at 40°C. Sections were washed in TBS, incubated in secondary antibody (1:600, Alexa Flour 647, Invitrogen) for 1 h at 40°C, counterstained with DAPI (ACD, CA) for 30 s and coverslipped with Prolong Gold mounting medium (Invitrogen).

### 5.6 In Situ Validation of snRNAseq

For *NLGN1* validation in OLs, images were acquired with a Zeiss LSM 710 NLO confocal microscope. A 1 mm^2^ area was montaged with a 40 × objective within the corpus callosum for each sample. Images were analyzed manually in FIJI where a quadrant grid was overlayed on the image. To obtain the percentage of *NLGN1* + OLs, two of the four quadrants were randomly selected using a random number generator and the number of *OLIG2*+ cells present within these areas were counted. These cells were classified as either *NLGN1* positive or negative depending on the expression of any *NLGN1* transcript, and the percentage of each type was recorded by a blinded observer. To quantify the amount of *NLGN1* expression within *OLIG2*+ cells, a random sampling of 120 *OLIG2*+ cells was selected and the amount of *NLGN1* mRNA puncta within each DAPI stained nucleus was manually counted and recorded for each animal using the cell counter plugin. To assess OL proximity to SMI312 labeled axons, a random sampling of 50 *OLIG2*+ cells were selected using a 6 × 6 grid and random number generator, and the distance from the center of the nucleus to the nearest axon was calculated using the measuring tool in FIJI. This distance was recorded and the presence or absence of *NLGN1* puncta was noted for each measurement. The distance values (μm) of *NLGN1* + and *NLGN1* − *OLIG2*+ cells were averaged across all sampling sites and animals within each group.

To qualitatively validate the expression of myelin markers within microglia, sections were treated with *MBP* and *PLP1* probes and immunolabeled with Iba1 antibody. Sections were imaged with confocal microscopy and a 40× objective to identify Iba1+ microglia with *MBP* and *PLP1* expression in the nuclear/perinuclear compartment.

### 5.7 Immunohistochemistry

To assess T cell infiltration into the brain, fixed brain sections from 24 male and female monkeys (12 control, 12 CR), were removed from −80°C, thawed at RT, and batch processed for immunohistochemistry. Fixed tissue from 9 of the 10 animals used for sequencing was included in this experiment, based on (Table 1a). Three sections containing the corpus callosum region of interest (ROI) were selected per subject. Sections were washed (3 × 5 min in 0.05 M Tris‐buffered saline [TBS] pH 7.4) to remove glycerol. Antigen retrieval was performed by microwaving sections in a tissue processor (PELCO Biowave, Ted Pella Inc. Redding, CA) at 550 W at 50°C for 10 min in 10 mM sodium citrate buffer (pH 6), followed by a 1‐h incubation at RT in the same buffer. Sections were washed and quenched in 10% H_2_O_2_ for 1 h to inactivate endogenous peroxidases, then blocked in Superblock for 1 h. Sections were incubated overnight at RT in anti‐CD3 primary antibody (1:500, Biorad, MCA1477) in TBS with 0.5% superblock, and 0.3% Triton‐X. The next day, sections were washed and incubated in biotinylated goat anti‐rat secondary antibody (1:600, BA‐9400, Vector Laboratories) in TBS with 0.5% superblock and 0.3% Triton‐X for 1 h. Sections were incubated in an avidin‐biotin complex (Vectastain ABC kit, Vector Laboratories, Burlingame, CA) for 1 h followed by 5 min incubation in a chromogen solution (0.5 mM 3–3‐diaminobenzadine with 0.03% hydrogen peroxide in TBS, Sigma‐Aldrich, St. Louis, MO). Sections were washed and mounted on gelatin‐coated slides and dried at RT for 48 h. Slides were dehydrated graded alcohols, incubated in xylene for 15 min and coverslipped with Permount (Fisher Scientific, Waltham, MA).

### 5.8 T Cell Quantification

Immunohistochemistry sections were imaged using the Huron Tissue Scope LE120 slide scanner at 40× magnification. Z stack images encompassing the entire frontal white matter region from three brain sections per animal was scanned. Image files were opened in FIJI and the entire corpus callosum was outlined (Figure 1d). As the size of the corpus callosum can vary slightly across animals, the area of the ROI was calculated for each animal (mm^2^) and recorded. Unbiased, exhaustive quantification of CD3^+^ cells was performed by a blinded observer using the Cell Counter plugin in FIJI. To ensure accuracy and consistency, a subset of sections was quantified by a second, blinded observer, and counts were considered valid if count variability fell within 10%. Only parenchymal CD3^+^ cells were counted whereas, perivascular CD3^+^ cells were excluded (Figure 5h) as we wanted to assess the number of CD3^+^ cells infiltrated into the parenchymal compartment. The number of parenchymal CD3^+^ cells identified were averaged across the 3 sections for each animal and recorded. Cell density was calculated using the area of counting frame from the 30 μM thick section.

### 5.9 Statistics

Data were analyzed using PRISM (GraphPad 10.2.2) statistical software. Multiple regression was performed using age as a continuous numerical variable and sex and diet group as categorical variables in all immunostaining experiments. Unpaired, two sample Students *t*‐tests were used to assess diet specific differences in *NLGN1* expression and T cell quantification. An *α ≤* 0.05 was set for all analyses. Simple linear regression was performed in PRISM using age as a continuous numerical variable and diet as categorical variable to assess T cell density across age.

## Author Contributions

Douglas L. Rosene and Chao Zhang obtained funding for the project. Julie A. Mattison and Kelli L. Vaughan maintained monkey colony and provided biological samples. Madelyn Ray and Ana T. Vitantonio designed sequencing workflow. Ana T. Vitantonio and Christina Dimovasili conducted tissue homogenization, nuclei extraction, and single nuclei library preparation. Yuchen Liu, Bingtian Ye, Jou‐Hsuan Roxie Lee, and Chao Zhang conducted computational analyses and visualization. Ana T. Vitantonio performed IHC, IF, and RNAscope experiments. Molly Hartigan, Benjamin Bouchard, and Bryce Conner provided technical assistance. Ana T. Vitantonio drafted the original manuscript. All authors participated in reviewing and editing the final manuscript for publication.

## Ethics Statement

This study was carried out in strict accordance with the recommendations in the Guide for the Care and Use of Laboratory Animals (National Research Council, 2011). All procedures were approved by the NIA Intramural Research Program's Institutional Animal Care and Use Committee.

## Conflicts of Interest

The authors declare no conflicts of interest.

## Supporting information


**Figure S1:** acel70298‐sup‐0001‐FigureS1.zip.

**Table S1:** Overview of quality control metrics.

## Data Availability

The sequencing data generated in this study are available at NCBI Sequence Read Archive (https://www.ncbi.nlm.nih.gov/sra) under accessions SRA: PRJNA1280011. Source code files for generating figures are available at: https://github.com/CZCBLab/Project_CRMetabolism.
