# Supplementary material for: Calorie Restriction Attenuates Transcriptional Aging Signatures in White Matter Oligodendrocytes and Immune Cells of the Monkey Brain
Source: Aging Cell. 2025 Nov 24;25(1):e70298. doi: 10.1111/acel.70298 (PMC12740093; doi:10.1111/acel.70298)
Supplement: Supplementary file 1 — Figure S1: acel70298‐sup‐0001‐FigureS1.zip. [file ACEL-25-e70298-s001.zip › acel70298-sup-0005-FigureS1-S4@Supplemental Fig 1-4 Captions.docx]

**Supplemental Figure 1. Differential Gene Expression in Oligodendrocytes**

Genes involved in mitochondrial metabolism are upregulated in CR OLs, including (A) Elk3 (log2FC = 0.371) and (B) Agk (log2FC = 0.424). Protein ubiquitination-related genes including (C) Klhl28 (log2FC = 0.427) and (D) Abtb2 (log2FC = 0.426) and cell adhesion genes (E) Mdga2 (log2FC = 0.374), (F) Nlgn1 (log2FC = 0.489), and (G) Ctnnd2 (log2FC = 0.613) also show upregulation in CR OLs. *p< 0.0001

**Supplemental Figure 2. Differential Gene Expression in Synaptic Oligodendrocytes**

(A,B) Glutamatergic receptor subunits Grid1 (log2FC = -0.351) and Grik2 (log2FC = -0.523) are downregulated in CR synaptic OLs. (C) Tmem117, is also downregulated in CR synaptic OLs (log2FC = -0.810). Synapse-related genes upregulated in CR synaptic OLs include (G) Nf1 (log2FC = 0.150, (E) Disc1 (log2FC = 0.354), and (F) Pick1 (log2FC = 0.421). Genes associated with protein turnover are also upregulated in CR synaptic OLs and include (G) Dnajc7(log2FC = 0.712), (H) Klh28 (log2FC = 0.601), and (I) Atg13 (log2FC = 0.460). Complement inhibitor, (J) Cd55, is upregulated in CR synaptic OLs (log2FC = 0.234). *p< 0.0001

**Supplemental Figure 3. T Cell Marker Genes are Uniquely Expressed by Microglia Subcluster 5** (A) T cell markers (*Themis, Skap1, Cd247, Cd96*) are not expressed in any other microglia subclusters. (B) T cell subcluster expresses CNS homing markers (*Itgal, Itga4*) (C) while lacking circulating, naïve T cell marker, *Ccr7*. (D) T cells and microglia subcluster 3 express T cell trafficking across the blood brain barrier (BBB) marker *Iqgap2*.

**Supplemental Figure 4. Microglia 4 enhance expression of lipid transport and metabolism genes**

Lipid transport and metabolism genes including (A) *ABCA2*, (B) *ABCA8*, (C) *SCARB1*, and (D) *SCARB2* are more highly expressed in Microglia 4 than in other microglia subtypes.
