# Supplementary figures and images for: Calorie Restriction Attenuates Transcriptional Aging Signatures in White Matter Oligodendrocytes and Immune Cells of the Monkey Brain
Source: Aging Cell. 2025 Nov 24;25(1):e70298. doi: 10.1111/acel.70298 (PMC12740093; doi:10.1111/acel.70298)

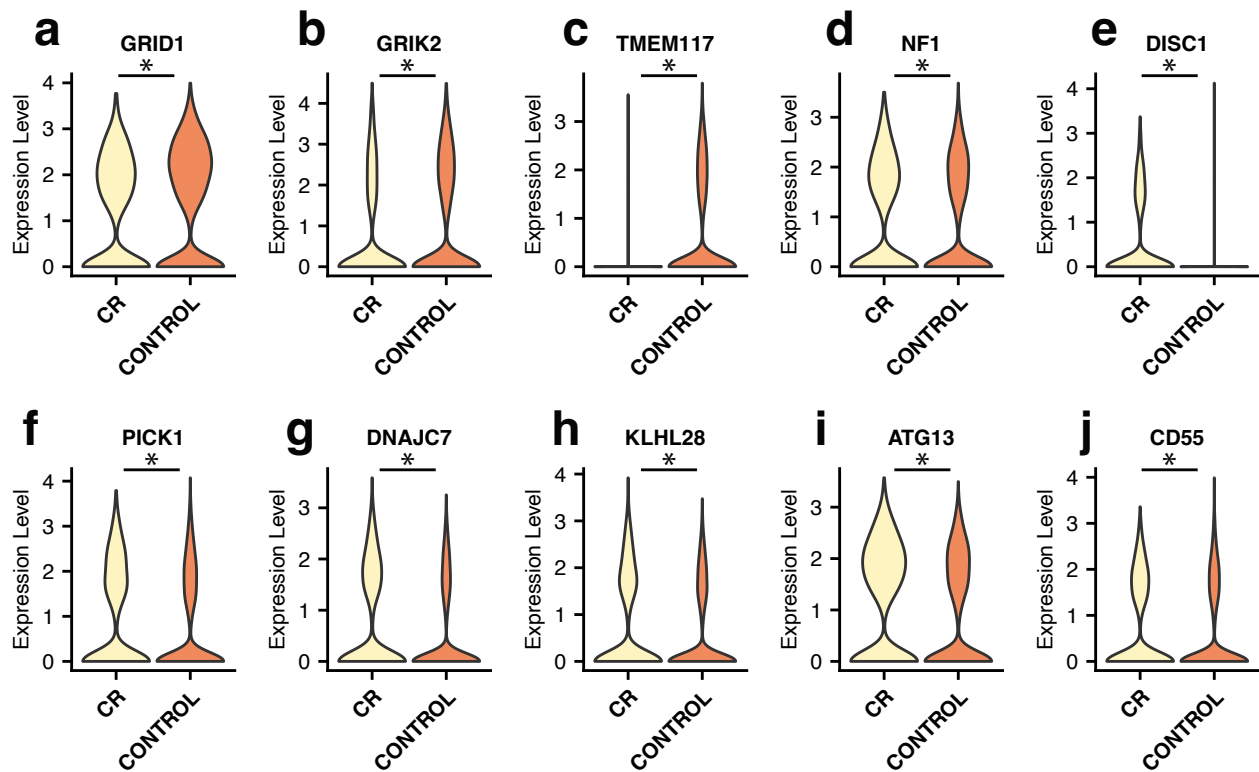

Supplement: Supplementary file 1 — Figure S1: acel70298‐sup‐0001‐FigureS1.zip. [file ACEL-25-e70298-s001.zip › acel70298-sup-0002-Supinfo2@S2.pdf]

Supplemental Figure 3

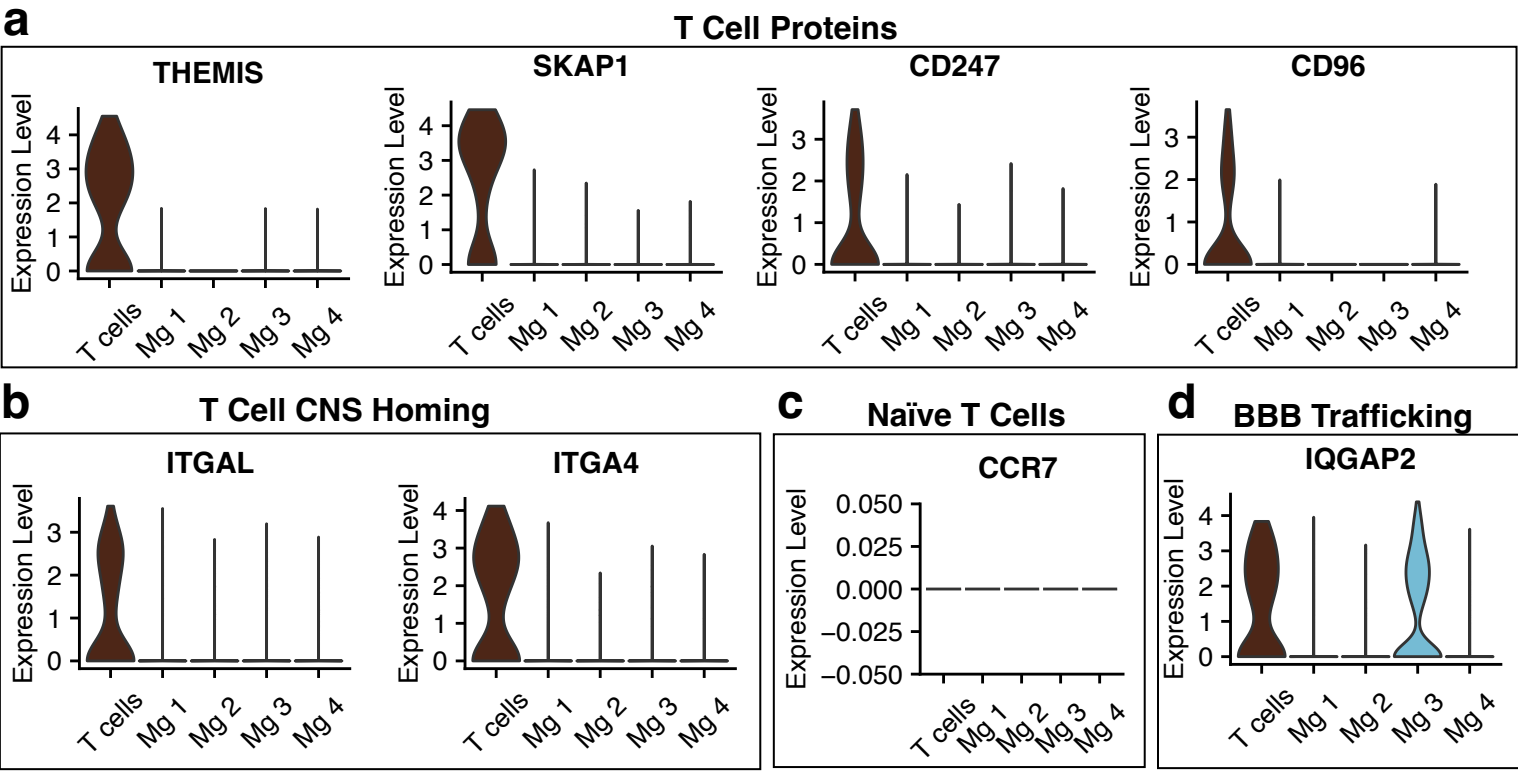

Supplement: Supplementary file 1 — Figure S1: acel70298‐sup‐0001‐FigureS1.zip. [file ACEL-25-e70298-s001.zip › acel70298-sup-0003-Supinfo3@S3_V1.pdf]

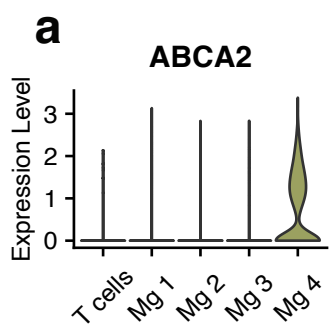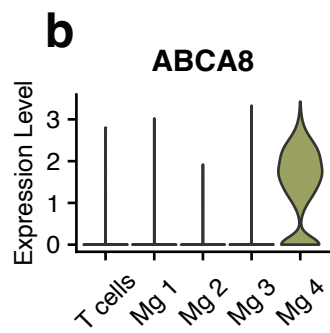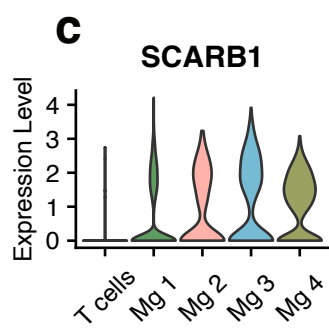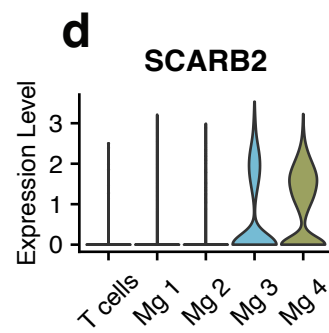

Supplement: Supplementary file 1 — Figure S1: acel70298‐sup‐0001‐FigureS1.zip. [file ACEL-25-e70298-s001.zip › acel70298-sup-0004-Supinfo4@S4.pdf]

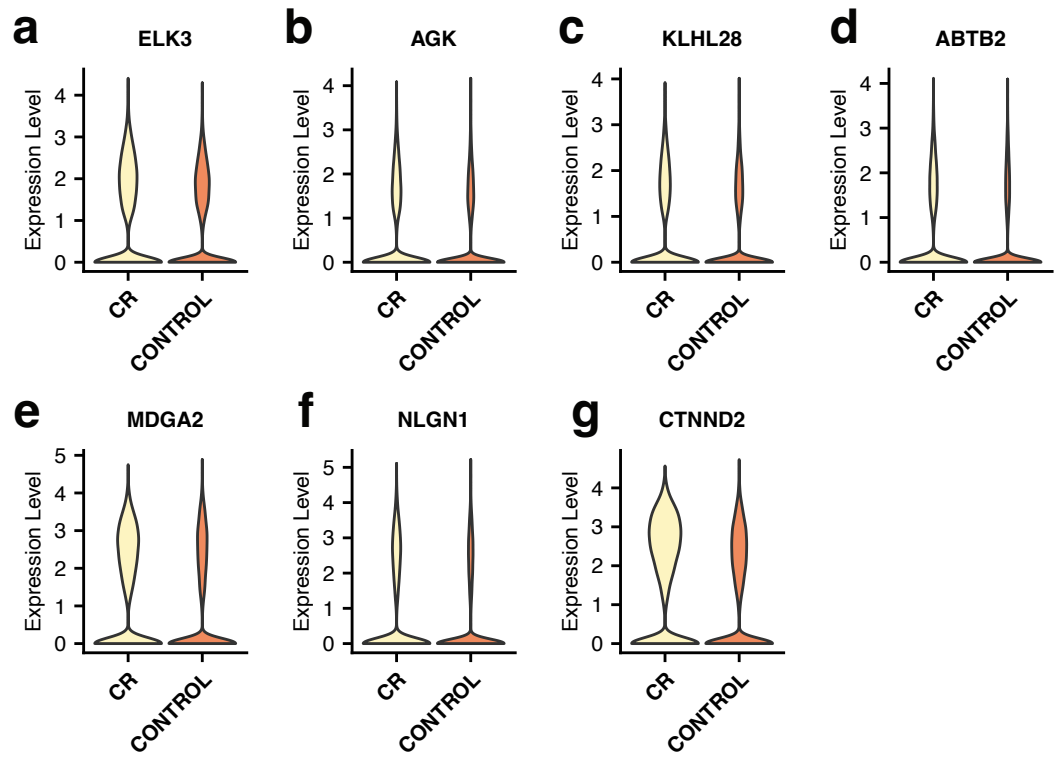

Supplement: Supplementary file 1 — Figure S1: acel70298‐sup‐0001‐FigureS1.zip. [file ACEL-25-e70298-s001.zip › acel70298-sup-0001-Supinfo1@S1.pdf]
