# Supplementary material for: Calorie Restriction Attenuates Transcriptional Aging Signatures in White Matter Oligodendrocytes and Immune Cells of the Monkey Brain
Source: Aging Cell. 2025 Nov 24;25(1):e70298. doi: 10.1111/acel.70298 (PMC12740093; doi:10.1111/acel.70298)
Supplement: Supplementary file 2 — Table S1: Overview of quality control metrics. [file ACEL-25-e70298-s002.docx]

**Supplementary Table 1. Overview of Quality Control Metrics**
